# As an unusual traumatic presentation, acetabular fracture and concomitant ipsilateral intertrochanteric femur fracture: a retrospective case series of 18 patients

**DOI:** 10.1186/s13018-020-02139-x

**Published:** 2020-12-09

**Authors:** Bo Liu, Wenhui Ma, Sikai Liu, Xiao Chen, Mengnan Li, Jia Huo, Huijie Li, Yongtai Han

**Affiliations:** Department of Osteonecrosis and Hip Surgery, The Third Hospital of Hebei Medical University, No.139 Ziqiang Road, Shijiazhuang, Hebei People’s Republic of China

**Keywords:** Fracture, Dislocation, Acetabulum, Intertrochanteric fracture

## Abstract

**Background:**

Acetabular fracture and concomitant ipsilateral intertrochanteric femur fracture has been suggested as an unusual traumatic presentation and rarely reported in the literature. The aims of this study were (1) to identify the etiological characteristics, (2) to summarize the treatment strategy, and (3) to present the mid- to long-term results of patients with this rare traumatic presentation.

**Methods:**

In this retrospective study, 18 patients (15 males, 3 females; mean age = 42.77 ± 17.74 years, range = 16 to 87 years) who were diagnosed and treated for simultaneous acetabular fracture and ipsilateral intertrochanteric fracture were included. Injury mechanisms, fracture classifications, and treatment strategies were noted. To assess functional status, the Harris score was used. To evaluate pain intensity, visual analogous scale (VAS) was used. The reduction quality of acetabular fractures was examined as per Matta’s standard. Postoperative complications were also recorded.

**Results:**

The mean follow-up was 10.04 ± 3.38 (range = 6.2 to 16 years). The most common injury mechanism was traffic accident, followed by falling from a tall height. As per the Evans classification, intertrochanteric fractures were defined as type 3 in 13 patients, type 2 in one patient, and type 4 in 4 patients. In acetabular fracture site, the most common fractures were posterior wall fractures, followed by anterior column fractures. All patients received internal fixation for their intertrochanteric fractures. Ten out of 18 patients also received internal fixation for their acetabular fracture. However, for the remaining patients, acetabular fractures were treated conservatively or with fracture fragment resection. Bony healing was achieved in all but one patient who died postoperatively. Twelve patients achieved excellent and good results (Harris score ≥ 80 points) whereas five patients achieved fair and poor results (Harris score < 80 points). The proportion of patients who achieved an excellent-good Harris score was 70.6%. Dislocation of the hip was found to be an independent risk factor (HR = 9.194, 95% CI = 1.024-82.515) for the poor patient outcome.

**Conclusion:**

To sum up, high-impact trauma is the main cause of acetabular fracture and concomitant ipsilateral intertrochanteric femur fracture. For patients who have undergone surgical treatment, fracture healing is usually achieved. However, the occurrence of complications, especially avascular necrosis, is the major cause of a poor prognosis. Dislocation of the hip joint at the time of injury is considered to be an important risk factor for a poor prognosis.

## Background

Fractures involving both the acetabulum and femoral trochanter have rarely been reported. The aetiology, treatment methods, and prognosis of this severe injury and the characteristics of patients with this injury remain unknown. Therefore, an investigation with a relatively large sample size needs to be conducted in patients with acetabular fractures and ipsilateral intertrochanteric fractures to help orthopaedic and traumatic surgeons manage such patients.

Generally, an acetabular fracture (especially a posterior wall fracture, the most common type of acetabular fracture) is usually caused by high-impact trauma, such as a car accident or a fall from a tall height [1, 2]. A so-called dashboard injury includes a fracture in the posterior wall of the acetabulum with or without anatomical insult to the proximal femur [3]. However, to date, only a few case reports of acetabular fractures combined with ipsilateral intertrochanteric fractures have been reported [4–6]. Browne et al reported three patients with this kind of injury [6]. Two of the injuries were caused by traffic accidents, and the other patient was injured via a fall from height. All three patients underwent open reduction and internal fixation (ORIF). However, the prognosis was not mentioned. Kuhn et al and Barrett et al separately reported similar cases [4, 5]. Both of the patients were injured in traffic accidents and were also treated with ORIF. However, owing to the limited sample size, the results might only reflect one or several aspects of the fracture characteristics. As already described by Mediouni et al., the goal of modern orthopaedic research is to fill the gap between basic sciences and clinical sciences, as well as contribute to translational orthopaedics [7–9]. To that end, we examined a case series of 18 patients with acetabular fractures combined with ipsilateral intertrochanteric fractures.

The aims of this study were (1) to identify the etiological characteristics, (2) to summarize the treatment strategy, and (3) to present the mid- to long-term results of patients with this rare traumatic presentation.

## Patients and methods

This study was approved by the Institutional Review Board of the Third Hospital of Hebei Medical University and was conducted in accordance with the Declaration of Helsinki. As this was a retrospective study and all patient information was deidentified before analysis, informed consent was only required for the patients whose radiological images were selected for publication. We retrospectively reviewed a total of 18 patients from 2003 to 2013. The inclusion criteria were patients who were treated in our hospital for combined ipsilateral acetabular fractures and intertrochanteric fractures. Patients were excluded if they had a history of old fractures involving the proximal femur or acetabulum, hip joint infections, or pathological fractures owing to a malignant disease. The general information, aetiology, and fracture classifications of the cases were identified based on either the patient’s radiological data or medical records. These data were collected and analysed by three authors (XC, ML, and JH). Evans’ classification system was used to evaluate the intertrochanteric fractures [3]. Moreover, the acetabular fracture sites were identified. The Arbeitsgemeinschaft fűr Osteosythesefragen/Orthopaedic Trauma Association (AO/OTA) classification system was also used to determine the fracture type of both the acetabular and femoral fractures [10].

### Treatments

All patients underwent surgical treatment for their fractures. The surgeries were performed by the same group of surgeons (YH, BL, WM, SL, and HL). All surgical treatments were performed electively when the patient’s haemodynamic status was stable. No emergency surgical treatments were performed. For some patients who had hip dislocation, reduction of the dislocated joint was performed at the same time as surgical treatment of the fracture. No emergency closed reductions were performed.

There were generally two types of surgical treatments: surgical treatment for acetabular fractures and surgical treatment for intertrochanteric fractures. In this study, the indications for surgical treatment for acetabular fractures were the presence of a large posterior wall fracture (affecting more than 30% of the area of the posterior wall), displacement exceeding 2 mm, an anterior or posterior column fracture, joint instability, and intraarticular free body formation. In other situations, for instance, an isolated posterior wall fracture (62-A1) without displacement exceeding 2 mm, conservative treatment might be sufficient for acetabular fractures. The most common surgical procedure for acetabular fractures is open reduction and internal fixation (ORIF). However, in some circumstances, the fracture fragment is too small to fix, and resection of the fragment is performed. In this study, all patients underwent surgical treatment (internal fixation) for the intertrochanteric fracture. If the acetabular fracture was surgically treated, the intertrochanteric fracture was reduced and fixed simultaneously with the same incision (or same operation) as was the acetabular fracture. If the acetabular fracture was treated conservatively, the intertrochanteric fracture was fixed with dynamic hip screws through the lateral approach or fixed with an intramedullary nail using a minimally invasive technique (closed reduction and internal fixation, CRIF).

Postoperatively, all patients received anti-thrombosis therapy. Non-weight-bearing movement of the hip joint was encouraged as soon as the pain could be tolerated. For the first 4 weeks, the patients were advised to avoid weight bearing on the affected limb. The patients attended 4 follow-ups (at 4 weeks, 12 weeks, 6 months, and 1 year) during the first year postoperatively. We used X-ray imaging to evaluate the bone healing conditions, and the X-ray scans were taken at the patients’ follow-ups. When callus formation was detected, the patient was allowed to perform partial weight-bearing (standing or walking with a cane or a walker). Full weight-bearing was allowed when the fracture was considered to exhibit a union.

### Outcome evaluation

The Harris score was used to evaluate the functional outcome [11]. The Harris scoring system consists of three parts: a pain assessment (44 points in total), functional assessment (51 points in total), and range of motion assessment (5 points in total). It has the advantages of high accuracy and good repeatability [11]. Pain was evaluated by using a 100-mm visual analogue scale (VAS) [12, 13]. Harris scores and VAS scores of patients were evaluated by three authors (XC, ML, and JH). The degree of pain was graded as follows: 1–3 points for mild pain, 4–6 points for moderate pain, and 7–10 points for severe pain. The reduction quality of the acetabular fracture was also investigated. The reduction quality was evaluated and analysed according to Matta’s standard [14], which was as follows: steps 0–2 mm indicated excellent-good reduction, steps 2–3 mm indicated fair reduction, and steps > 3 mm indicated poor reduction. The duration of bone healing and incidence of complications were also investigated [11]. If conversion to total hip arthroplasty (THA) was performed in a patient, the time to THA and the Harris score before THA were also recorded. A patient was considered to have poor outcomes if the Harris score was less than 80 points or if the patient underwent conversion to THA.

### Statistical analysis

Statistical analyses were performed using the statistical software SPSS, version 19.0 for Windows (IBM, Armonk, New York). The continuous variables were expressed as the mean ± SD (range), and the categorical variables were expressed as frequencies. A paired *t* test was used to compare the Harris score between the affected limb and the opposite limb. Cox regression analyses were used to assess the association between potential risk factors and poor outcomes in patients. Owing to the limited sample size, we first built univariable Cox regression models to find the potential risk factors. Then, a multivariable Cox regression model was built to find the independent risk factors for poor outcomes. A *p* value less than 0.05 was considered significant.

## Results

### General information

A total of 18 patients were initially included in this study. The mean age of the patients was 42.77 ± 17.74 years (range from 16 years to 87 years). There were 15 males and 3 females. In this study, injuries occurred most commonly via traffic accidents. Falling from a tall height was the second most common cause of injury, followed by a crushing event. Only one elderly (87 years) female patient was injured by slipping and falling on the ground. Six patients had posterior dislocation of the hip joint. All patients received surgical treatment, and the mean time to surgery was 8.72 ± 5.37 days (range from 3 days to 23 days). In terms of comorbidities, one patient had acute respiratory distress syndrome (oxygenation index = 130 mmHg) and was treated with mechanical ventilation in the intensive care unit. Traumatic shock was identified in two patients, who were treated with fluid resuscitation (including transfusion) and vasoactive agents. One patient had a history of pneumonia. Multiple rib fractures and pneumohemothorax were identified in one patient, who was treated conservatively. Seven patients had other fractures; there was a pubic fracture in five patients (treated conservatively), fractures of the ulna and radius in one patient (treated by open reduction and internal fixation), and a clavicle fracture in one patient (treated conservatively). Except for the patient who had a history of pneumonia, the comorbidities of all other patients were cured without significant aftereffects. The follow-up time was 6.2 to 16 years. Details of the patients’ general characteristics are shown in Table 1.

**Table 1:** General characteristics of the patients and fracture classifications

Case	Age (years)	Sex	Side	Aetiology	Dislocation of hip	Time to surgery (days)	Evans classification for intertrochanteric fracture (AO/OTA classification)	Classification for acetabular fracture (AO/OTA classification)	Follow-up time (years)
1	49	Male	Right	Crushing injury	No	8	Type IV (31-A2)	Posterior Wall (62-A1)	16.0
2	46	Male	Left	Crushing injury	No	14	Type III (31-A2)	Transverse & Posterior Wall (62-B1)	15.7
3	46	Male	Right	Fall from a tall height	No	8	Type III (31-A2)	Anterior Column (62-A3)	15.4
4	31	Female	Right	Crushing injury	Yes	3	Type III (31-A2)	Anterior Column (62-A3)	13.6
5	26	Male	Left	Traffic accident	No	5	Type III (31-A1)	Posterior Wall (62-A1)	12.0
6	42	Male	Right	Fall from a tall height	No	4	Type III (31-A2)	Posterior Wall (62-A1)	11.4
7	25	Female	Left	Traffic accident	Yes	12	Type III (31-A1)	Posterior Wall (62-A1)	10.0
8	69	Male	Left	Traffic accident	No	6	Type III (31-A1)	Posterior Wall (62-A1)	9.5
9	87	Female	Left	Slip, trip, or fall	No	15	Type III (31-A2)	Posterior Wall (62-A1)	-
10	47	Male	Right	Traffic accident	No	5	Type IV (31-A2)	Posterior Wall (62-A1)	9.3
11	22	Male	Right	Traffic accident	Yes	5	Type III (31-A2)	Transverse & Posterior Wall (62-B1)	7.8
12	47	Male	Left	Traffic accident	Yes	8	Type IV (31-A2)	Posterior Wall (62-A1)	8.0
13	42	Male	Left	Traffic accident	No	6	Type II (31-A1)	Posterior Wall (62-A1)	7.7
14	39	Male	Right	Traffic accident	Yes	4	Type III (31-A1)	Posterior Wall (62-A1)	7.8
15	16	Male	Left	Traffic accident	No	4	Type IV (31-A2)	Posterior Column (62-A2)	7.2
16	43	Male	Right	Traffic accident	No	15	Type III (31-A1)	Posterior Wall (62-A1)	6.6
17	28	Male	Right	Fall from a tall height	Yes	12	Type III (31-A2)	Posterior Wall (62-A1)	6.4
18	65	Male	Right	Fall from a tall height	No	23	Type III (31-A1)	Anterior Column (62-A3)	6.2

Note: The patient in case 9 died because of pulmonary infection after the operation

### Treatments

For acetabular fractures, 6/18 patients underwent conservative treatment, 2/18 patients underwent resection of the fracture fragments, and 10/18 patients underwent open reduction and internal fixation (ORIF). Nine of 12 patients underwent ORIF or resection via the Kocher-Langenbeck approach. In addition, the ilioinguinal approach, Watson-Jones approach and Smith-Peterson approach were used for the other three patients (Table 2). According to Matta’s standard, excellent or good reduction was achieved in 14 patients, and fair reduction was achieved in 3 patients [14]. There was one patient with poor fracture reduction. In terms of treatment for the intertrochanteric fractures, twelve of the eighteen intertrochanteric fractures were reduced and fixed via the same incision (or same operation) as that used for the acetabular fracture, 2/18 were reduced and fixed via the lateral approach, and 4/18 were reduced and fixed via the minimally invasive approach (closed reduction and internal fixation). According to the type of internal fixation, 5/18 intertrochanteric fractures were treated by a dynamic hip screw (DHS), 9/18 intertrochanteric fractures were treated by an intramedullary nail, and 4/18 intertrochanteric fractures were treated by a plate and screws (Table 2).

**Table 2:** Treatment methods performed among the patients

Case	Acetabular fracture			Intertrochanteric fracture		
	Treatment	Approach	Internal fixation	Treatment	Approach	Internal fixation
1	ORIF	Kocher-Langenbeck	Screws	ORIF	Same incision for acetabular fracture	DHS
2	ORIF	Kocher-Langenbeck	Plate with screws	ORIF	Same incision for acetabular fracture	DHS
3	Conservative	-	-	ORIF	Lateral approach	DHS
4	ORIF	Ilioinguinal approach	Plate with screws	ORIF	Same incision for acetabular fracture	DHS
5	Conservative	-	-	ORIF	Lateral approach	Intramedullary nail
6	ORIF	Watson-Jones	Plate with screws	ORIF	Same incision for acetabular fracture	DHS
7	ORIF	Kocher-Langenbeck	Screws	ORIF	Same incision for acetabular fracture	Plate with screws
8	Conservative	-	-	CRIF	-	Intramedullary nail
9	Conservative	-	-	CRIF	-	Intramedullary nail
10	ORIF	Kocher-Langenbeck	Plate with screws	ORIF	Same incision for acetabular fracture	Intramedullary nail
11	ORIF	Kocher-Langenbeck	Plate with screws	ORIF	Same incision for acetabular fracture	Plate with screws
12	Resection	Kocher-Langenbeck	-	ORIF	Same incision for acetabular fracture	Intramedullary nail
13	Conservative	-	-	CRIF	-	Intramedullary nail
14	ORIF	Kocher-Langenbeck	Plate with screws	ORIF	Same incision for acetabular fracture	Plate with screws
15	ORIF	Kocher-Langenbeck	Plate with screws	ORIF	Same incision for acetabular fracture	Intramedullary nail
16	Resection	Smith-Peterson	-	ORIF	Same incision for acetabular fracture	Intramedullary nail
17	ORIF	Kocher-Langenbeck	Screws	ORIF	Same incision for acetabular fracture	Plate with screws
18	Conservative	-	-	CRIF	-	Intramedullary nail

*ORIF* open reduction and internal fixation, *CRIF* closed reduction and internal fixation, *DHS* dynamic hip screw

### Prognosis

One elderly (87 years) female patient had a pulmonary disease and died at 24 days postoperatively because of an uncontrollable pulmonary infection (therefore, we excluded this patient from our study cohort when analysing the prognosis and its influencing factors). In the other patients, all the fractures demonstrated bony healing. In 13/17 patients, fracture union was identified at 12 weeks postoperatively. In the other 4/17 patients, fracture union was identified at 24 weeks postoperatively.

The average visual analogue scale score and Harris score at the final follow-up (or before arthroplasty in the patients who underwent conversion to total hip arthroplasty) were 1.71 ± 2.08 points (range from 0 points to 6 points) and 84.12 ± 11.72 points (range from 55 points to 96 points), respectively. The average Harris score was significantly lower for the affected limb than for the opposite limb (84.12 ± 11.72 vs. 99.18 ± 1.07, *t* = − 5.196, *p* < 0.001). There were 12 patients with excellent or good results (Harris score ≥ 80 points) and 5 patients with fair or poor results (Harris score < 80 points). The proportion of patients with an excellent or good Harris score was 70.6%. Three patients underwent conversion to total hip arthroplasty (THA). The time from the initial treatment to THA was 1.8 years, 2.2 years, and 2.5 years for these three patients. The prognoses of the patients are shown in Table 3.

**Table 3:** Prognosis and complications of the patients at the final follow-up

Case	Reduction quality^b^	Bone healing time (weeks)	Harris score	VAS score	Heterotopic ossification (Brooker grade)	Posttraumatic arthritis (Kellgren-Lawrence grade)	Avascular necrosis of femoral head	Converted to THA	Time to THA
1	Excellent-good	12	92	0	-	0	No	No	-
2	Fair	12	85	1	-	1	No	No	-
3	Excellent-good	12	89	1	-	1	No	No	-
4	Excellent-good	24	91	0	-	0	No	No	-
5	Excellent-good	24	88	0	-	0	No	No	-
6	Excellent-good	12	96	0	3	0	No	No	-
7	Poor	24	79	4	-	3	No	No	-
8	Excellent-good	12	93	1	-	0	No	No	-
9	-	-	-	-	-	-	-	-	-
10	Excellent-good	12	93	0	1	1	No	No	-
11^a^	Fair	12	55	5	-	1	Yes	Yes	2.2
12^a^	Excellent-good	12	67	6	1	2	Yes	Yes	1.8
13	Excellent-good	24	91	0	-	0	No	No	-
14	Excellent-good	12	84	1	-	2	No	No	-
15	Fair	12	96	0	1	0	No	No	-
16^a^	Excellent-good	12	66	5	3	1	Yes	Yes	2.5
17	Excellent-good	12	77	3	2	1	Yes	No	-
18	Excellent-good	12	88	2	-	1	No	No	-

Note: The patient in case 9 died because of pulmonary infection after the operation

*VAS* visual analogue scale, *THA* total hip arthroplasty

^a^Last follow-up before total hip arthroplasty

^b^Matta’s standard for acetabular fractures

Three kinds of complications were commonly identified in the patients with acetabular fractures and ipsilateral intertrochanteric fractures. Heterotopic ossification (HO) was identified in 6/17 patients. Two cases were classified as Brooker grade 3, and four cases were classified as Brooker grades 1–2. Ten of 17 patients were found to have signs of posttraumatic arthritis. There was one patient with arthritis of Kellgren-Lawrence grade 3 and 9 patients with arthritis of Kellgren-Lawrence grades 1–2. Avascular necrosis was identified in four patients. Three of these patients underwent conversion to THA (Table 3).

**Fig. 1:**
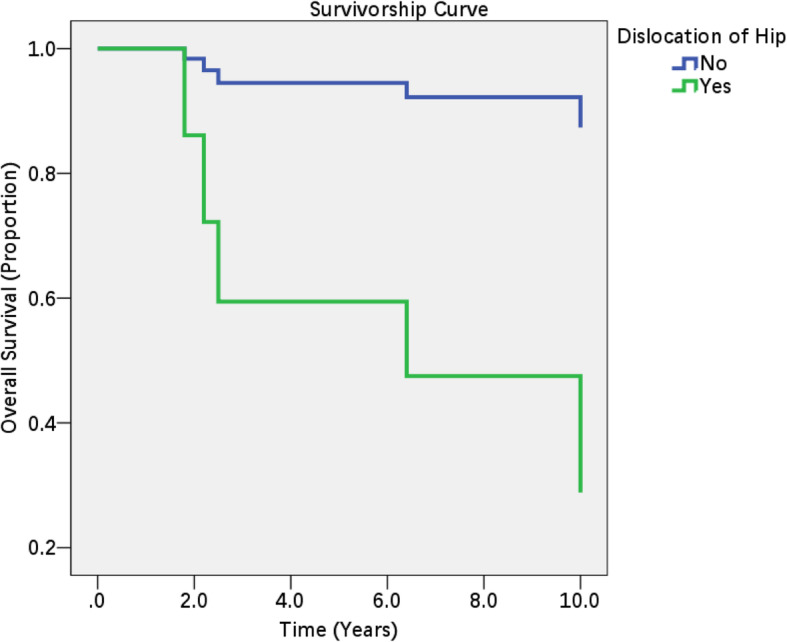
Survivorship curve for patients with an acetabular fracture and ipsilateral intertrochanteric fracture. Note: One elderly (87 years) female patient was excluded from our study cohort because she had a chronic pulmonary disease and died of pulmonary infection at 24 days postoperatively. Patients were considered have a poor outcome if they had a Harris score less than 80 points or underwent total hip arthroplasty

Cox regression models were built to identify the potential risk factors for poor patient outcomes. The univariate Cox regression models revealed that only hip dislocation was a potential risk factor (Table 4). Then, a multivariate Cox regression model was built to identify the independent risk factors. Stepwise regression revealed that only dislocation of the hip was an independent risk factor (HR = 9.194, 95% CI = 1.024–82.515) for poor outcomes in the patients (Table 5). The survivorship curve is shown in Fig. 1.

**Table 4:** Univariate Cox regression models for the potential risk factors for poor outcomes in the patients

Risk factors		HR	95% CI	*p*
Age (years)		0.959	0.890–1.033	0.269
Sex	Male (Ref.)			
	Female	1.293	0.139–11.994	0.821
Side	Left (Ref.)			
	Right	1.066	0.178–6.399	0.994
Aetiology	Traffic accident (Ref.)			
	Other	0.234	0.025–2.201	0.204
Dislocation of the hip	No (Ref.)			
	Yes	9.194	1.024–2.515	0.048
Time to surgery (days)		1.091	0.936–1.270	0.266
Evans classification for intertrochanteric fracture	2 or 3(Ref.)			
	4	1.005	0.111–9.057	0.997
Acetabular fracture site	Posterior wall (Ref.)			
	Other	0.443	0.049–3.987	0.468
Treatment for the acetabular fracture	Conservative or Resection (Ref.)			
	ORIF	0.828	0.137–5.005	0.837
Reduction quality of the acetabular fracture	Excellent-good (Ref.)			
	Fair-poor	2.002	0.333–12.043	0.448

Note: For patients who underwent conversion to total hip arthroplasty, the survival time is the period between the time of fracture occurrence and the time of total hip arthroplasty. Survival events were defined in patients who did not undergo total hip arthroplasty and had a Harris score higher than 80 points at the last follow-up

*HR* hazard ratio, *CI* confidence interval, *ORIF* open reduction and internal fixation

**Table 5:** Multivariate Cox regression models for the potential risk factors for poor outcomes in the patients (only the variables in the equation are shown in the table)

Risk factors		HR	95% CI	*p*
Dislocation of hip	No (Ref.)			
	Yes	9.194	1.024–82.515	0.048

Note: Covariates including age, aetiology, dislocation of hip, and time to surgery were initially entered into the equation. By stepwise regression, only dislocation of the hip was a certain risk factor

*HR* hazard ratio, CI confidence interval

## Discussion

### General characteristics

Fractures involving both the acetabulum and ipsilateral femoral trochanter have rarely been reported [15]. Generally, the patients in this study had several of the same characteristics. First, almost all the patients were victims of high-impact trauma (e.g., a traffic accident or a fall from a tall height). Comorbidities such as dislocation of the hip joint, traumatic shock, and fractures at other sites were commonly identified in these patients. Second, for the patients with hip joint dislocation, closed reduction was not successful. The integrity of the femur had been compromised. Third, surgical interventions were necessary for all intertrochanteric fractures. However, for acetabular fractures, conservative treatment was performed for certain cases. Finally, most patients demonstrated acceptable hip joint function (Harris score > 80 points) after undergoing open reduction and internal fixation treatment. However, some patients, especially those who had concomitant hip joint dislocation, had a poor prognosis.

### Treatments

Because they result from high-impact, intra-articular injuries, acetabular fractures with ipsilateral intertrochanteric fractures are innately difficult for surgeons to manage [4, 6, 16, 17]. In this study, instead of an emergency surgery, all patients underwent an operation several days after the injury, when he or she was haemodynamically stable and well prepared for surgery. In the studies by Kuhn et al. and Barrett et al., the periods from the injury to surgery were 4 days and 5 days, respectively [4, 5]. As in our study, this delay was introduced to wait for the patient’s physiologic condition to stabilize.

For acetabular fractures, surgical treatment is not always necessary because some fractures are considered stable without displacement, and the fracture has little influence on the function of the hip joint during weight-bearing [18–21]. The type of treatment selected is also influenced by the surgeon’s experience and preference. In 1980, Browne et al. reported three similar patients. In all of them, the acetabular fractures were treated conservatively. However, in two recent reports, all the acetabular fractures were treated surgically with open reduction and internal fixation. In this study, when a fracture was considered unstable with displacement exceeding 2 mm, surgical treatment was performed to achieve anatomical reduction and prevent the development of posttraumatic arthritis [22–24]. The results showed that most patients achieved excellent or good reduction of the acetabular fractures. This might be the reason why the incidence of post-traumatic arthritis was relatively low in our study. Reconstruction plates and screws were the most commonly used internal fixation tools for acetabular fractures.

The treatment principle for an intertrochanteric fracture combined with an acetabular fracture is similar to that of an isolated intertrochanteric fracture. Intertrochanteric fractures are generally considered unstable; thus, surgical treatment is performed in all patients [3, 25]. If a patient receives surgical treatment for an acetabular fracture, the intertrochanteric fracture can be treated by the same incision (or with an extension). Otherwise, the intertrochanteric fracture can be treated with a minimally invasive surgical procedure (closed reduction and internal fixation). In the past, dynamic hip screws were commonly used for intertrochanteric fracture fixation, and more recently, intramedullary nails and plates (with screws) have been commonly used. By the surgical or conservative treatment described above, both acetabular and intertrochanteric fractures achieved bony healing in all patients.

### Prognosis

In this study, the majority of patients achieved excellent or good hip function (Harris score ≥ 80 points). Unfortunately, there were still 5 patients with poor outcomes. From the results, we found that the onset of complications, especially avascular necrosis, is the main cause of poor clinical outcomes.

**Fig. 2:**
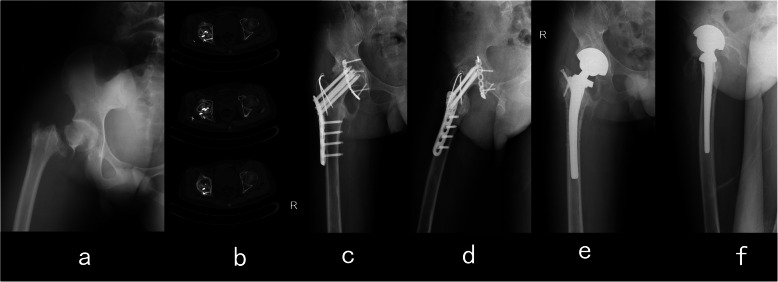
Case 11. A 22-year-old male patient had a traumatic acetabular fracture combined with an ipsilateral intertrochanteric fracture in his right hip. The patient underwent open reduction and internal fixation surgery for both his acetabulum and proximal femur. Then, 2.2 years after surgery, avascular necrosis of the right femoral head was detected, and revision with total hip arthroplasty was performed. **a** Anterior-posterior view immediately after injury. **b** Computed tomography image showing avascular necrosis of the right femoral head. **c** Anterior-posterior view before hip arthroplasty. **d** Lateral view before hip arthroplasty. **e** Anterior-posterior view after hip arthroplasty. **f** Lateral view after hip arthroplasty

**Fig. 3:**
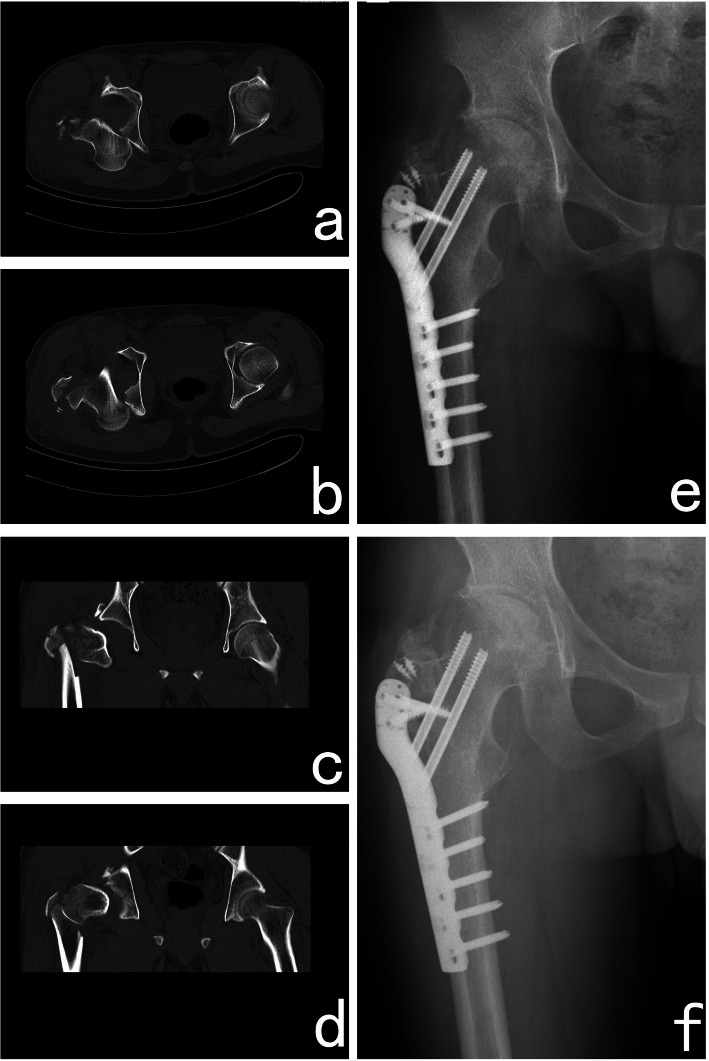
Case 17. A 28-year-old male patient had a traumatic acetabular fracture combined with an ipsilateral intertrochanteric fracture in his right hip. The patient underwent open reduction and internal fixation surgery for both his acetabulum and proximal femur. An absorbable screw was used to fix the fracture fragment of the posterior wall. Plates and screws were used to fix the intertrochanteric fracture. **a**–**d** Computed tomography image showing a posterior wall fracture of the acetabulum and dislocation of the hip joint. **e** Anterior-posterior view at the final follow-up. **f** Lateral view before hip arthroplasty at the final follow-up

It is well known that an isolated acetabular fracture can cause avascular necrosis of the femoral head, but the incidence rate is relatively low [26, 27]. In a recent study, the incidence of avascular necrosis was 5.6% after a traumatic acetabular fracture [11] (Figs. 2 and 3). However, in this study, the incidence of avascular necrosis was extraordinarily high. Furthermore, in our Cox regression models, dislocation of the hip joint was identified as the sole independent risk factor for poor outcomes. When the fractures were combined with a dislocated hip joint, the patient was not treated with emergency closed reduction because the continuity of the femur was damaged, thus making closed reduction quite difficult to perform. On the basis of the results of some similar studies and our results [4–6], we suspect that when the hip joint is not reduced, the blood supply of the femoral head might be affected, causing avascular necrosis and leading to poor clinical outcomes. Although some recent studies have shown that early reduction of the dislocated hip joint does not have a large favourable impact in terms of avascular necrosis [1, 15, 28], the results of our study showed that delayed reduction might be a cause of poor outcomes that cannot be dismissed. Hence, rapid reduction of the hip joint should be considered in the future to prevent avascular necrosis.

In addition, we found that most cases of avascular necrosis are identified within 3 years after the injury.

### Limitations

This study has several limitations. First, this is a retrospective study with a small sample size, so some important information and potential risk factors may not have been accounted for. Second, with severe injuries such as the fractures examined herein, patients often have additional injuries, but we did not account for any additional injuries in the analysis. Third, we found that hip dislocation was an independent risk factor for poor patient outcomes. However, all cases of dislocation of the hip joint were reduced several days after injury; thus, we cannot determine whether early reduction is helpful for reducing the incidence of avascular necrosis and further improving the prognosis of patients.

## Conclusions

To sum up, high-impact trauma is the main cause of acetabular fracture and concomitant ipsilateral intertrochanteric femur fracture. For patients who have undergone surgical treatment, fracture healing is usually achieved. However, the occurrence of complications, especially avascular necrosis, is the major cause of a poor prognosis. Dislocation of the hip joint at the time of injury is considered to be an important risk factor for a poor prognosis.

## Data Availability

All data generated or analysed during this study are included in this published article.

## Abbreviations

ORIF: Open reduction and internal fixation
CRIF: Closed reduction and internal fixation
VAS: Visual analogue scale
THA: Total hip arthroplasty
DHS: Dynamic hip screw
HO: Heterotopic ossification
HR: Hazard ratio
CI: Confidence interval
